# The Prevalence of ST-Segment Elevation Myocardial Infarction in Patients Presenting in the Emergency Service of Galati Hospital from 2015 to 2019

**DOI:** 10.3390/clinpract14040114

**Published:** 2024-07-16

**Authors:** Liliana Dragomir, Virginia Marina, Aurelian-Dumitrache Anghele, Mihaela Anghele, Cosmina-Alina Moscu

**Affiliations:** 1 Faculty of Medicine and Pharmacy, “Dunarea de Jos” University of Galati, 800008 Galati, Romania; lilianadragomir2017@gmail.com (L.D.); anghele_aurelian@yahoo.com (A.-D.A.); cosmina_caluian@yahoo.com (C.-A.M.); 2 Medical Department of Occupational Health, Facultatea de Medicina si Farmacie, “Dunarea de Jos” University, 800008 Galati, Romania; 3 Clinical-Medical Department, Faculty of Medicine and Pharmacy, Dunărea de Jos University of Galati, 800201 Galati, Romania; mihaela.anghele@ugal.ro

**Keywords:** cardiovascular disease, air transport, prevalence, ST-elevation myocardial infarction

## Abstract

Background and Objectives: The purpose of this study is to determine the prevalence of cardiovascular emergencies and the relationships between these emergencies and the personal medical histories of patients. Materials and Methods: This study is retrospective, observational, and longitudinal, spanning five years from 1 January 2015 to 31 December 2019. Descriptive elements were observed and recorded to conduct statistical analysis on the cardiovascular characteristics of 723 patients transported by air and treated at the Emergency County Hospital of Galati, Romania. Results: Cardiovascular disease is a complex condition that often originates in the heart and presents with a variety of symptoms. Deaths related to cardiovascular diseases outnumber cancer-related deaths in both men and women worldwide. The one-year mortality rate for patients admitted to the hospital with acute pulmonary edema can be as high as 40%. Coronary heart disease is the leading cause of death and disability in the Western world and globally. Conclusions: The highest prevalence of cardiovascular diseases was noted in 2016, particularly among elderly men, who appear to be more affected by these conditions, while liver disease was minimal. In our study, the most prevalent cardiovascular disease was ST-elevation myocardial infarction. Gender plays a role in the risk of cardiovascular emergencies, with men being at a higher risk of developing life-threatening conditions. Additionally, there is a linear increase in risk with age for developing these pathologies.

## 1. Introduction

Cardiovascular diseases have a complex pathophysiology and can manifest through various symptoms, with the heart being the primary starting point. According to Eurostat statistics, cardiovascular diseases are the leading cause of death in the European Union. Baltic countries, including Romania, have recorded the highest percentage (50–60%) [1].

The most significant gender differences were observed in the Baltic countries, Romania, and Slovenia, where the proportion of women dying from circulatory diseases was 11.1 and 15.4 percentage points higher than that of men.

An international study predicts a life expectancy of 80.8 years for women and 74.8 years for men. Cardiovascular disease deaths in all countries far exceed cancer deaths for both sexes. Among the 15 European Society of Cardiology member countries, cancer accounts for more deaths than cardiovascular diseases in men in fifteen countries and in women in five countries [1]. 

Overall, the age-adjusted prevalence of hypertension decreased from 47.0% in 1999–2000 to 41.7% in 2013–2014, and then it increased to 45.4% in 2017–2018 [2].

Among men, the age-adjusted prevalence followed a similar pattern, decreasing from 51.7% in 1999–2000 to 45.2% in 2013–2014, and then increasing to 51.0% in 2017–2018. However, there was no significant change in the age-adjusted prevalence of hypertension among women from 1999–2000 (42.0%) to 2017–2018 (39.7%).

Acute pulmonary edema is a medical emergency that requires immediate treatment. It is characterized by dyspnea and hypoxia due to fluid accumulation in the lungs, affecting gas exchange and lung compliance [3]. The one-year mortality rate for patients admitted to the hospital with acute pulmonary oedema is up to 40% [4]. Common causes include myocardial ischemia, arrhythmias (atrial fibrillation), acute valvular dysfunction, fluid overload, pulmonary embolism, renal artery stenosis, non-adherence to treatment, and adverse drug effects [5].

Although rare, acute aortic dissection is a catastrophic condition: caused by the separation of the aortic wall layers. A tear in the intimal layer leads to the progression of the dissection, mainly due to blood leakage between the intima and media layers. Acute aortic dissection is associated with very high mortality rates, with most patients dying before reaching the Emergency Department.

Coronary heart disease is the leading cause of death and disability in the Western world and globally. Prolonged myocardial oxygen deprivation can result in myocardial cell death and necrosis [6]. Symptoms may include chest discomfort or pressure radiating to the neck, jaw, shoulder, or arm. According to the Atherosclerosis Risk in Communities (ARIC) study conducted by the National Heart, Lung, and Blood Institute (NHLBI), between 2005 and 2014, there were an estimated 605,000 new myocardial infarctions and 200,000 recurrent myocardial infarctions annually [7]. 

This study aims to determine the prevalence of ST-segment elevation myocardial infarction, pulmonary edema and hypertensive emergencies in Galați, Romania, based on individual variables and their relationship with the patients’ medical histories.

## 2. Material and Methods

### 2.1. Study Population and Study Protocol

The present study is a retrospective, observational, and longitudinal study conducted over a 5-year period, from 1 January 2015 to 31 December 2019. It aimed to observe and record all descriptive elements to perform statistical analyses based on the subjective characteristics of the group.

A total of 723 patients, aged between 5 and 94 years, with cardiovascular pathologies, were studied. They were investigated and treated at the Emergency County Hospital in Galati, Romania.

The inclusion criteria for the study required patients to have cardiovascular pathologies as the main diagnosis, admission dates within the study period, and the use of aerospace transport. Exclusion criteria included patients with no information about air transport from the accident site, those without cardiovascular diseases, and those lacking information about treatment used.

All information collected from the observation forms was entered into sampling lists and centralizing tables and included basic diagnosis, clinical data on the status of vital functions, therapeutic approach, and personal medical history. Information on patient behavior like smoking, alcoholism, and smoking was also recorded. Variability was maintained in terms of background, with both urban and rural patients included. The study was conducted in accordance with the World Medical Association Declaration of Helsinki using a protocol approved by the local Bioethics Committee, and hospital administration approval was obtained. (Protocol code: 1594/21.01.202)

### 2.2. Data Analysis

The final data analysis was conducted in Microsoft Excel 2019 and IBM SPSS Statistics version 26.0 to study causal relationships and correlations between the variables described later in this paper.

For efficient analysis of the research batch, descriptive statistical indicators were calculated and analyzed for all variables where this calculation approach was considered useful (amplitude, dispersion, standard deviation, root mean square deviation and dispersion, Kurtosis index, location indicators, including mean, median, minimum, and maximum value).

Furthermore, 95% confidence intervals were also determined for the variables studied to statistically assess the proportions.

In terms of analytical statistics, numerous tests were applied to determine the regression model, with the Chi-square (χ^2^) test used to determine whether a difference between observed data and expected data is random or due to a relationship between the variables studied. Bivariate correlations were analyzed using the Pearson correlation coefficient, with a significance threshold of *p* = 0.05.

## 3. Results

### 3.1. Socio-Demographic Analysis of the Studied Group

Out of the total number of our patients, 72.89% were male. The mean age of the studied subjects was 61.12 years, with a standard deviation of 0.46 years and a median age value of 62. The minimum age recorded in the group was 5 years, and the maximum age was 94 years.

When analyzing the group by age groups, the highest proportion was recorded among the elderly at 59.72%, followed by middle-aged adults at 35.72%. Young adults represented a small percentage of the studied group (4.28%), and only 0.28% of the subjects were children. Most subjects were not active smokers at the time of assessment, accounting for 95.17%, while only 4.83% of subjects claimed tobacco use (see Table 1).

A total of 60.22% of patients were smokers, while 39.78% were non-smokers.

Additionally, most female subjects did not have alcoholism at the time of examination, with 31.63% of subjects not having this risk factor and 68.37% having it.

### 3.2. Prevalence of Cardiovascular Pathology

Following the statistical analysis performed on the study group, the highest prevalence was found in 2016, and 25.69% of the cases studied were from this year. A quasi-symmetry can also be observed between the years 2017 and 2018, where 21.69% of cases were registered in 2017 and 21.56% were registered in 2018.

In 2019, the prevalence of cardiovascular pathologies was only 16.44%, and the lowest prevalence in this study group was recorded in 2015; only 14.64% were from this period (Table 2).

In terms of distribution by age group, it can be seen that the highest prevalence of cardiovascular pathologies was found in 2016 among the elderly, representing 15.19% of the cases studied. Also, the prevalence among the elderly was high in all years studied; thus, 7.73% of subjects were reported in 2015, 14.23% in 2017, 13.12% in 2018, and 9.39% in 2019 (Table 2).

Regarding the correlation between patient gender and the incidence of cardiovascular pathologies, throughout the study period of this paper, there were almost constant prevalences among males, with a higher number of incidences of cardiovascular pathologies compared to females (Table 3).

Another important socio-demographic factor is background. For our study, we observed, according to the statistical results, a higher predisposition of urban people to cardiovascular pathology compared to rural areas. A correlation between the environment of origin and the sex of the subjects revealed that men showed an incidence about twice as high in urban areas compared to rural areas. As for females, no major differences were observed between the urban and rural backgrounds, with the number of patients being almost symmetrical for rural and urban. Distribution of the batch according to background and gender of the subjects (Table 3).

### 3.3. Prevalence of Cardiovascular Pathologies and Statistical Relationship with Personal Pathological History

In our study, we observed that ST-segment elevation myocardial infarction, accounted for 80.41% of the total number of cases included in the group, hypertension accounted for 10.64%, cardio-respiratory arrest for only 2.35%, aortic dissection for 2.07%, and pulmonary thromboembolism for 1.52% (Table 4).

The study group exhibited a nearly symmetrical distribution of the prevalence of hypertension, with 50.35% of subjects presenting the condition while 49.65% did not. Upon evaluating the frequency of a history of diabetes mellitus in the studied group, it was observed that the majority of subjects (85.77%) did not have this pathology at the time of the initial observation.

Another pathology frequently associated with cardiac dysfunction is liver disease. The majority of the subjects did not have liver disease at the time of examination (98.07%), with the most common condition being hypercholesterolemia (1.10%). One subject had cirrhosis associated with esophageal varices (0.17%), one had biliary lithiasis (0.14%), and one had hypertriglyceridemia (0.14%).

Within our study group, 3.73% of patients had associated neurological pathologies. The highest incidence was for stroke (2.49%), followed by epilepsy (0.28%). The remaining cases were pathological associations of stroke and epilepsy, epilepsy and cavernoma, stroke and cavernoma, Alzheimer’s, Parkinson’s, ALS, and meningioma in almost equal percentages (Table 5).

It should be mentioned that the group of patients studied is part of the category of cardiovascular emergencies, which benefited from initial aeromedical intervention, stabilization, transport, and then admission to the Emergency Department of the Galati County Hospital. Regarding pharmacological therapy, it should be noted that the following data only refer to the drugs administered at pick-up and during air transport to the Emergency Department (Table 5). 

### 3.4. Analysis of Subjects’ Vital Parameters and Their Evolution

The mean value of initial heart failure is 85.47, with a standard deviation of 0.774. A significant positive correlation was observed between initial heart’ failure and evolving heart’ failure, with a correlation of 0.68, indicating a large effect size (*p* < 0.001, 95.00% CI = [0.63, 0.72]). The mean baseline systolic blood pressure was 143.84, with a standard deviation of 33.421. The correlation result was examined based on an alpha value of 0.05. A significant positive correlation was observed between initial systolic blood pressure and evolving systolic blood pressure, with a correlation of 0.666, indicating a large effect size (*p* < 0.001, 95.00% CI = [0.62, 0.71]). The mean baseline diastolic blood pressure was 87.05, with a standard deviation of 20.681, noting a significant positive correlation between initial diastolic blood pressure and evolving diastolic blood pressure, with a correlation of 0.61, indicating a large effect size (*p* < 0.001, 95.00% CI = [0.56, 0.66]). The mean initial partial saturation in oxygen (SpO2) was 96.57, with a standard deviation of 6.114 (Table 6).

The mean serum glucose value recorded was 191.53, with a standard deviation of 87.459. Additionally, the minimum recorded serum glucose number was 78 mg/dL, while the maximum number was 500 mg/dL.

The statistical relationship between the diagnosis of the patients and their underlying pathologies was evaluated to infer a causal relationship between them. Following Chi-square test analysis, the following variables were not significant: diabetes mellitus (alpha value of 0.05, χ^2^(8) = 14.64, *p* = 0.067); cardiac spectrum pathology (alpha value of 0.05, χ^2^(432) = 275.11, *p* = 1.000); neurological disorders (alpha value of 0.05, χ^2^(72) = 80.42, *p* = 0.232).

Chi-square test results were significant based on an alpha value of 0.05, χ^2^(16) = 123.33, *p* < 0.001, suggesting that the variables diagnosis and psychiatric pathology are statistically significantly related. Also, the variables diagnosis and liver pathology show a statistically significant dependence relationship (alpha of 0.05, χ^2^(48) = 111.32, *p* < 0.001).

To determine the causal relationship between the risk factors analyzed and the subjects’ diagnosis, statistical dependence tests were developed between the present diagnosis and the risk factors. Chi-square test results were not significant for the variable defined by the presence of smoking (*p* = 0.346), and ethylic defined by a *p*-value = 0.475.

## 4. Discussion

This localized study in Galati, Romania, describes the age, gender, and origin distribution of patients admitted to the Emergency Department with cardiovascular emergencies. Details concerning the underlying pathologies at the time of admission were also analyzed.

The mean age of the study subjects was 61.12, reflecting the senior population. Cardiovascular emergencies were more common in metropolitan regions than in rural areas, possibly due to better medical care accessibility. The most prevalent pathology, was ST-segment elevation myocardial infarction (80.41%). A minimal incidence of underlying pathologies (diabetes mellitus, cardiac pathology, neurological pathology, psychiatric pathology, and hepatic pathology) was observed.

Chi-square tests of independence were used to determine the relationship between the primary diagnosis and the underlying pathologies. Significant results were found only with hepatic (χ^2^(48) = 111.32, *p* < 0.001) and psychiatric pathologies (χ^2^(16) = 123.33, *p* < 0.001).

The study found the highest prevalence of cardiovascular pathologies (ST segment elevation myocardial infarction) in 2016, particularly among elderly individuals. Men appeared to be more affected by cardiovascular pathologies. Urban areas had a higher incidence of cardiovascular pathology. No significant relationship was identified between aerospace transport and underlying pathologies (Table 7).

Unfortunately, it was not possible to demonstrate a causal relationship between the severity of pathologies and the environment of origin. Therefore, it could not be objectively determined if the use of aerospace transport among urban subjects was due to the increased incidence of severe pathologies or whether the incidence was higher due to the higher number of subjects coming from this environment.

Mortality associated with acute coronary syndromes remains high, especially in individuals with extensive myocardial damage who are at a high risk of adverse outcomes [1,2,3].

Prognostic scores in acute coronary syndromes are utilized to assess the risk of major complications, stratify patients based on risk, and determine the most appropriate therapeutic approach, which may include the need for percutaneous angioplasty (PCI) or coronary artery bypass grafting (CABG).

The Global Registry of Acute Coronary Events (GRACE) risk score is a widely validated tool recommended for risk stratification in patients presenting with ACS, as outlined in international guidelines [4,6].

For patients presenting without persistent ST-segment elevation, short-term risk assessment using the GRACE score can help identify high-risk individuals who would benefit from early invasive management in addition to optimal pharmacologic therapy [5,7].

The GRACE risk score is essential for international ACS guidelines to determine the timing of interventional strategies and estimate prognosis in patients with NSTE-ACS, while also allowing appropriate risk assessment for patients with ST-elevation myocardial infarction [5,8,9].

Pre-hospital time can be significantly reduced because helicopters have higher speeds and more direct routes than ground ambulances, thus avoiding traffic.

The independent economic analysis of Helicopter Emergency Medical Services concluded that “in general, patients transported by helicopter showed benefits in terms of survival, time to reach the health unit, time to final treatment, better outcomes, or an overall benefit” [10].

According to a study by Wright JT Jr., which included 9361 patients aged over 50 years with high cardiovascular risk but without diabetes, they had a lower incidence of a combined cardiovascular endpoint [11].

For both men and women, a similar pattern of increasing prevalence of hypertension by age was observed. The prevalence of hypertension was higher among men than women aged 18–39 years [3].

Frequent comorbidities included obesity, dyslipidemia, and low blood glucose.

The age- and gender-specific prevalence of three or more comorbid diseases among male patients with hypertension was significantly higher than those in the 30–59-year-old age group [12].

The prevalence of pulmonary edema is significantly higher in patients with heart failure [13]. This is a distressing condition, with a discharge rate to 1-year survival rate of 50%. The six-year follow-up mortality rate was 85% in patients with congestive heart failure.

Men are usually affected more than women, and older people are at higher risk of developing pulmonary edema [14].

I recall an extremely important study in which cases of AD diagnosed between 2005 and 2012 were identified in the Taiwan National Health Insurance (NHI) comprehensive research database [14]. A total of 9092 individuals with a mean age of 64.4 ± 15.1 years were identified. Patients who received drug therapy were older compared to patients who required invasive procedures for aortic dissection. In addition, patients in group C were associated with more comorbidities than the other two groups. Patients in group B associated more frequently in their medical history, chronic obstructive pulmonary disease (11.36%) and coronary artery disease (18.62%), unlike those in group A, who achieved results of 8.59% and 15.47%, respectively [14].

The mean age was 52.0 years old in the entire cohort. Multiple comorbidities were more common in the older age groups (60 years, 70 years, and 80 years), while patients in the 20 year-old age group had the highest proportion of Marfan syndrome at 28.1% [15]. 

The results revealed that as age increased, there was an increasing rate of comorbidities, including diabetes, renal failure, and previous stroke.

Older patients with ST segment elevation myocardial infarction present have a higher mortality rate compared to younger patients. Additionally, they are at a higher risk of acute aortic syndrome [16].

In addition to older age, being female is also considered a risk factor for adverse outcomes following the repair of acute type A aortic dissection [17].

In conclusion, morbidity and mortality rates are similar between patients under and over 75 years old after the repair of an acute type A aortic dissection. Female patients under 75 years had a higher in-hospital mortality rate compared to their male counterparts [10,18].

The aforementioned risk factors were significantly associated with acute myocardial infarction, with the exception of alcohol consumption, which showed a weaker association. Smoking and an abnormal apolipoprotein ratio had the strongest association with acute myocardial infarction. The increased risk associated with diabetes and hypertension was found to be higher in women, while the protective effects of exercise and alcohol were also greater in women [19].

Some non-modifiable risk factors for myocardial infarction include older age, male gender (men tend to experience myocardial infarction at a younger age), and genetics (there is an increased risk of myocardial infarction if a first-degree relative has a history of cardiovascular events before the age of 50) [20].

Another risk factor in the occurrence of acute myocardial infarction or other coronary accidents is represented by the consumption of recreational drugs with significant effects on cardiovascular function [21].

Morphine and heroin, derived from its semi-synthesis, are the most frequently used narcotic analgesics, representing almost half of the deaths due to drug use. Various bradyarrhythmias and tachyarrhythmias have been reported [22].

In this sense, a study carried out over a period of 3 years, in which a number of 609 patients were followed who needed medical care as a result of single or multiple poisoning, in the Emergency Department of the Galati County Clinical Hospital, Romania, observed that the most frequent intoxications were with ethnobotanicals for 407 patients, followed by the consumption of heroin (70 patients), and other drugs were MDMA (17 patients), cocaine (24 patients), amphetamines (28 patients), cannabis (10 patients), and ecstasy (35 patients), a high percentage requiring hospitalization in the intensive care unit, one of the reasons being the compromise of cardiovascular function [23].

The goals of pharmacotherapy are to reduce morbidity and prevent complications [24].

The behavior of patients who try to hide their conditions can represent an important lesson for clinical practice [25].

Despite many advances in treatment, acute myocardial infarction still carries a high mortality rate, with most deaths occurring before hospital arrival. Patients who had non-ST-elevation myocardial infarction received coronary stents, were treated with conservative medical management, or were referred for coronary artery bypass surgery [26].

The overall prognosis depends on the degree of heart muscle damage and ejection fraction, with patients who have preserved left ventricular function tending to have better outcomes [27].

Researchers found that chest pain has a lower predictive value for obstructive coronary artery disease in women compared to men, especially in younger women. This was attributed to the relatively high prevalence of non-obstructive coronary artery disease in the latter group. Women with acute coronary stenosis, non-ST segment elevation myocardial infarction, or ST segment elevation myocardial infarction often do not have major coronary stenosis on angiography, unlike men. Women with non-obstructive coronary artery disease typically present with atypical angina with pain that may be more intense and persist for at least 30 min [28].

Advancements in medical therapy and coronary interventions have significantly reduced mortality rates [29].

The presence of instability or neuroticism appears to increase the likelihood of experiencing burnout syndrome [30].

Addressing emotional exhaustion is critical for improving job satisfaction. Encouraging interpersonal support and creating positive work and life experiences can help prevent burnout and improve individual well-being [31].

To evaluate and guide treatment for patients with acute coronary syndromes, particularly acute myocardial infarction, the Syntax II score is a useful tool. It combines clinical and anatomical information to provide a comprehensive assessment of the risks and benefits of revascularization interventions such as percutaneous coronary angioplasty or coronary artery bypass surgery, ensuring personalized treatment to enhance patient outcomes [32].

### Limits of the Research

This study was limited to a small group of patients due to the complexity of the statistical tests. Therefore, Chi-square independence tests were not always statistically significant, as the test’s outcome was influenced by the group size. Real-time data collection was not feasible, so the data analyzed relied on the observation sheet records.

Despite these limitations, this study paves the way for future large-scale, nationwide, and pathology-specific studies.

To highlight the significance of this research, we have drawn correlations with existing international studies. Cardiovascular emergencies remain a global medical priority, underscoring the importance of focusing on these conditions.

Preventative measures for cardiovascular disease should be emphasized by healthcare professionals, including educating patients about cardiovascular risks and promoting lifestyle changes and risk factor management.

## 5. Conclusions

The incidence of cardiovascular disease is higher in older people, with an increased prevalence each year studied in this paper. The present study, which covers a small number of subjects (representing major-risk cardiovascular emergencies), shows an almost linear trend in the incidence of cardiovascular pathologies over the period studied, following the national trend.

The most frequent cardiovascular pathology is ST-segment elevation myocardial infarction. In order to obtain an acceptable time interval, comply with the recommendation of specialized guidelines to reach a PCI center within 120 min and reduce the primary intervention time, it is necessary to use airway resources. This proves its usefulness both from the point-time factor perspective and from the medical team’s standpoint.

Vicious behaviors, such as smoking and alcohol consumption, in the subjects of the present study are closely causally related to the development of acute ST-segment elevation, myocardial infarction, and hypertension. Harmful behaviors involving the use of high-risk drugs or new psychoactive substances severely compromise cardiac function, precipitating often irreversible complications.

A positive linear correlation exists between the baseline parameters and their progression.

Gender plays a role in influencing the risk of cardiovascular emergencies, with men being at a higher risk of developing life-threatening conditions, likely due to the prevalence of cardiovascular risk factors.

While a direct relationship between multiple underlying pathologies and the primary diagnosis was not established, this does not rule out their potential association with the development of cardiovascular emergencies. Further research is needed to explore potential causal relationships.

The development of preventive and personalized primary medicine in the field of cardiovascular diseases is a significant challenge, but it could bring about major changes in healthcare. Therefore, it is necessary to develop long-term projects to encourage research in this field and to facilitate the earliest possible adoption of this type of medicine in clinical practice.

## Figures and Tables

**Table 1:** Socio-demographic characteristics of subjects.

Gender	Male	Percentage	Female	Percentage
	527	72.89%	196	27.11%
Age				
Children (0–18 years)	2	0.28%	0	0
Young adults (19–39 years)	21	2.89%	10	1.39%
Middle-aged adults (40–59 years)	147	20.27%	112	15.45%
Seniors > 60 ani	357	49.23%	74	10.49%
Smokers				
Yes	436	60.22%	168	85.72%
No	91	39.78%	28	16.68%
Ethylic				
Yes	457	63.11%	134	68.37%
No	70	36.81%	62	31.63%
Environment				
Urban	378	71.73%	149	20.99%
Rural	149	28.27%	123	17.50%

**Table 2:** Prevalence of cases in the studied years and distribution by ages.

Year	Cardiovascular Pathologies		Children		Young Adults		Middle-Aged Adults		Seniors	
	N°	%	N°	%	N°	%	N°	%	N°	%
2015	106	14.64	1	0.14	4	0.55	45	6.22	56	7.73
2016	186	25.69	0	0	5	0.69	71	9.81	110	15.19
2017	157	21.69	0	0	8	1.1	46	6.35	103	14.23
2018	156	21.55	0	0	9	1.24	52	7.18	95	13.12
2019	119	16.44	1	0.14	5	0.69	45	6.22	68	9.39

**Table 3:** Gender and background distribution of the batch by years of study.

Year	Male			Female		
	Number	Urban	Rural	Number	Urban	Rural
2015	76	26	40	30	16	14
2016	127	90	37	58	31	27
2017	116	89	27	41	22	19
2018	120	88	32	36	19	18
2019	88	57	31	31	16	15
	527	344	183	196	108	88

**Table 4:** Distribution of personal pathological history in the studied group during 2015–2019.

Type of Pathologies	Number of Patients	Percentage %
Hypertension	364	50.35
Sudden death	6	0.83
Cardiorespiratory arrest	17	2.34
Acute aortic dissection	15	2.07
Natural accident	3	0.41
Pulmonary thromboembolism	11	1.52
ST elevation myocardial infarction	583	80.41
Road accident	10	1.38
Trauma	3	0.41
Diabetes mellitus	103	14.23
Neurological disorders	9	3.73
Psychiatric disorders	721	99.59
Hepatic pathologies		
Acute hepatitis	1	0.14
Chronic hepatitis	2	0.28
Hepatic cirrhosis	1	0.14
Esophageal varices	1	0.14
Gallstones	1	0.14
Hypertriglyceridemia	1	0.14
Hypercholesterolemia	8	1.10
Neurological disorders		
Stroke	18	2.5%
Epilepsy	2	0.3%
Stroke and epilepsy	1	0.1%
Epilepsy and cavernoma	1	0.1%
Stroke and cavernoma	1	0.1%
Alzheimer’s disease	1	0.1%
Parkinson’s disease	1	0.1%
Amyotrophic lateral sclerosis	1	0.1%
Meningioma	1	0.1%

**Table 5:** Distribution by drug classes used for patients studied.

Drug Classes	Type of Drug	Patient’s Number	Percentage %
Antiarrhythmics Class IB	Na Channel blockers	46	6.34
Antiarrhythmics Class III	K Channel blockers	20	2.76
	Selective beta-channel blockers	5	0.69
	Cardiac glycosides	3	0.41
Opioid Analgesics	Morphine	15	2.07
	Fentanyl	30	4.14
	Tramadol	1	0.14
Non-Opioid	Paracetamol	72	9.94
Inotropic and Vasopressor Agents	Adrenaline	10	1.38
	Dopamine	30	4.14
	Noradrenaline	1	0.14
	Adrenaline and noradrenaline	3	0.41
	Adrenaline, noradrenaline, and dopamine	2	0.28
Antihypertensives	Alpha-blockers	4	0.55
	Angiotensin-converting enzyme inhibitors	41	5.66
Vasodilators	Glyceryl trinitrate	166	22.90
Anticoagulants	Unfractionated heparin	362	44.97
	Enoxaparin sodium	100	13.8
Parenteral Diuretics	Furosemide	28	3.86
	Osmofundin 15%	3	0.41
	Mannitol 20%	7	0.97

**Table 6:** Correlations between initial and evolving vital parameters.

		GCS Initial	GCS Evolving
Glasgow Coma Scale initial	Pearson Correlation	1	0.850 **
	Sig. (2-tailed)		0.000
	N	722	558
GCS evolving	Pearson Correlation	0.850 **	1
	Sig. (2-tailed)	0.000	
	N	558	561
Systolic blood pressure initial	Pearson Correlation	1	0.666 **
	Sig. (2-tailed)		0.000
	N	722	572
Systolic blood pressure evolving	Pearson Correlation	0.666 **	1
	Sig. (2-tailed)	0.000	
	N	572	573
Diastolic blood pressure initial	Pearson Correlation	1	0.603 **
	Sig. (2-tailed)		0.000
	N	718	568
Diastolic blood pressure evolving	Pearson Correlation	0.603 **	1
	Sig. (2-tailed)	0.000	
	N	568	571
Heart’s failure initial	Pearson Correlation	1	0.676 **
	Sig. (2-tailed)		0.000
	N	706	531
Heart’s failure evolving	Pearson Correlation	0.676 **	1
	Sig. (2-tailed)	0.000	
	N	531	545
SpO2 initial	Pearson Correlation	1	0.321 **
	Sig. (2-tailed)		0.000
	N	710	541
SpO2 evolving	Pearson Correlation	0.321 **	1
	Sig. (2-tailed)	0.000	
	N	541	550

** Correlation is significant at the level (2-tailed).

**Table 7:** Distribution by ST segment elevation myocardial infarction-based study group.

Age Groups	Number of Patients	Percentage %
Young adults	24	4.1%
Middle-aged adults	211	36.2%
Seniors	348	59.7%
Gender		
Male	429	73.6%
Female	153	26.2%
Missing	1	0.2%
Year		
2015	84	14.4%
2016	145	24.9%
2017	134	23.0%
2018	123	21.1%

## Data Availability

No new data were created or analyzed in this study.
